# Effect of *Triticum turgidum* subsp. *turanicum* wheat on irritable bowel syndrome: a double-blinded randomised dietary intervention trial

**DOI:** 10.1017/S000711451400018X

**Published:** 2014-02-13

**Authors:** Francesco Sofi, Anne Whittaker, Anna Maria Gori, Francesca Cesari, Elisabetta Surrenti, Rosanna Abbate, Gian Franco Gensini, Stefano Benedettelli, Alessandro Casini

**Affiliations:** 1 Agency of Nutrition, Careggi University Hospital, Florence, Italy; 2 Department of Experimental and Clinical Medicine, University of Florence, Largo Brambilla 3, Florence50134, Italy; 3 Don Carlo Gnocchi Foundation Florence, Florence, Italy; 4 Interdipartimental Center for Research on Food and Nutrition, University of Florence, Florence, Italy; 5 Department of Agrifood Production and Environmental Sciences, University of Florence, Florence, Italy; 6 Digestive Pathophysiology and Motility Unit, Careggi University Hospital, Florence, Italy

**Keywords:** Irritable bowel syndrome, Grains, Wheat, Diets, Cytokines

## Abstract

The aim of the present study was to examine the effect of a replacement diet with organic, semi-whole-grain products derived from *Triticum turgidum* subsp. *turanicum* (ancient) wheat on irritable bowel syndrome (IBS) symptoms and inflammatory/biochemical parameters. A double-blinded randomised cross-over trial was performed using twenty participants (thirteen females and seven males, aged 18–59 years) classified as having moderate IBS. Participants received products (bread, pasta, biscuits and crackers) made either from ancient or modern wheat for 6 weeks in a random order. Symptoms due to IBS were evaluated using two questionnaires, which were compiled both at baseline and on a weekly basis during the intervention period. Blood analyses were carried out at the beginning and end of each respective intervention period. During the intervention period with ancient wheat products, patients experienced a significant decrease in the severity of IBS symptoms, such as abdominal pain (*P*< 0·0001), bloating (*P*= 0·004), satisfaction with stool consistency (*P*< 0·001) and tiredness (*P*< 0·0001). No significant difference was observed after the intervention period with modern wheat products. Similarly, patients reported significant amelioration in the severity of gastrointestinal symptoms only after the ancient wheat intervention period, as measured by the intensity of pain (*P*= 0·001), the frequency of pain (*P*< 0·0001), bloating (*P*< 0·0001), abdominal distension (*P*< 0·001) and the quality of life (*P*< 0·0001). Interestingly, the inflammatory profile showed a significant reduction in the circulating levels of pro-inflammatory cytokines, including IL-6, IL-17, interferon-γ, monocyte chemotactic protein-1 and vascular endothelial growth factor after the intervention period with ancient wheat products, but not after the control period. In conclusion, significant improvements in both IBS symptoms and the inflammatory profile were reported after the ingestion of ancient wheat products.

Irritable bowel syndrome (IBS) is a chronic gastrointestinal disorder with recurrent abdominal discomfort or pain, in combination with altered bowel habits, affecting approximately 10–20 % of the general population^(^ ^1^ ^,^ ^2^ ^)^. Patients often experience abdominal pain/discomfort and bloating derived from the intake of food items based on wheat and dairy products^(^ ^3^ ^)^. However, the relative importance of the role played by different food items and food groups in the generation of IBS symptoms is not well defined, and current dietary advice is largely based on known effects of nutrients on gut physiology rather than on controlled trials^(^ ^3^ ^)^.

IBS is a disorder with both high direct and indirect costs^(^ ^1^ ^,^ ^2^ ^)^. For the majority of individuals suffering from IBS symptoms, where conventional treatment is often ineffective and patients often report a significant reduction in the quality of life, an investigation into the role of diet as an alternative strategy for symptom relief is of great importance^(^ ^3^ ^)^. Moreover, the inclusion of functional foods in the diet remains one of the simplest and potentially most attractive options, and has the additional potential for increasing ‘healthy food’ markets. Recently, research interest has been focused on ancient wheat grain varieties as a rich source of functional health-promoting substances^(^ ^4^ ^)^. Among the ancient wheat grain varieties, *Triticum turgidum* subsp. *turanicum* is emerging as one of the most potentially interesting types of wheat in both distribution and marketing sectors.

In our previous study^(^ ^5^ ^)^, a replacement diet with products made from such ancient wheat has been shown to induce significant improvements in the inflammatory profile of a healthy population. Given recent findings indicating that IBS is a low-grade inflammation condition associated with changes in cytokine profiles^(^ ^6^ ^,^ ^7^ ^)^, the working hypothesis of the present study was that a replacement diet with ancient wheat products might similarly result in improved cytokine levels. The study by Dinan *et al.* ^(^ ^8^ ^)^ has been considered instrumental in linking changes in cytokine levels with IBS symptoms, and has shown that a medicine-induced increase in the level of the pro-inflammatory cytokine IL-6 is associated with increased abdominal pain/discomfort and bloating. The aim of the present intervention study was to test the efficacy of a replacement diet with *T. turgidum* subsp. *turanicum* (ancient) wheat products on gastrointestinal symptoms of IBS in relation to inflammatory cytokine and biochemical parameters.

## Materials and methods

### Study population

Patients were recruited between September and November 2012 through advertisements among the outpatients of Careggi University Hospital in Florence. Participants were considered eligible for inclusion if they were aged between 18 and 65 years and met the Rome III criteria for IBS diagnosis. Coeliac disease was excluded by serological examinations and by the absence of human leucocyte antigen (HLA)-DQ2 and HLA-DQ8 haplotypes. Exclusion criteria were as follows: inflammatory bowel disease, any known organic disease or clinical alarm signs; pregnancy, breast-feeding or lactose intolerance; BMI >35 or < 18 kg/m^2^ (suggesting an abnormal diet or health status). Individuals reporting a recent change in IBS medication (in the preceding month) were excluded in order to avoid the likelihood of co-intervention bias. Written informed consent was obtained from each participant before the initial screening visit and before randomisation.

### Wheat varieties

Organically grown *T. turgidum* subsp. *turanicum* wheat, given by Kamut International Limited and obtained from Saskatchewan, Canada, the principal cultivation area, was utilised as the ancient wheat under investigation. The seed was milled by Molino Silvestri, and both semi-whole wheat semolina and flour were obtained. As the modern wheat, semi-whole wheat semolina and wheat flour, respectively, derived from a mix of modern Italian durum and soft wheat varieties were obtained from the same mill. As with the ancient wheat, both the Italian soft and durum wheat varieties, hereafter referred to as the modern wheat, were similarly cultivated under organic agricultural conditions. To further standardise the comparison, all transformation preparation procedures were identical for both the ancient (experimental) and modern wheat under study. Pasta was prepared from the ancient and modern durum wheat by Pastificio Artigiano Fabbri s.a.s., an artisan pasta-maker, whereas bread, biscuits and crackers were made from the ancient and modern soft wheat by the artisan enterprise of Panificio Menchetti Pietro di Santi e Figli s.n.c.

### Study protocol

Patients were randomised according to a computer-generated list of random numbers. The present study was a randomised, double-blinded cross-over trial. Baseline symptom data were collected during a 2-week run-in period. After the run-in period, eligible participants were randomly divided into two groups (*n* 10 individuals per group), each assigned to consume first either the ancient or modern wheat products, respectively. Starting from November 2012, participants in both groups received 500 g pasta/week, 150 g bread/d, 500 g crackers/month and 1 kg biscuits/month for a period of 6 weeks. During the intervention period, all participants were encouraged to maintain their ‘normal eating habits’, but they were not permitted to eat other cereal grain products. A washout period of 6 weeks was then implemented, in which participants were permitted to eat all foods according to their ‘normal eating habits’. From February 2013, the second intervention period was implemented with the group, assigned to consume the modern wheat products in the first intervention period, now assigned to consume the ancient wheat products, and vice versa. The institutional review board at the University of Florence approved the study protocol.

At baseline and at weeks 6, 12 and 18, all subjects were examined between 07.00 and 09.30 hours after a 12 h fasting period. Furthermore, subjects were asked to avoid strenuous physical activity during the day before the examination. On arrival, weight was determined to the nearest 100 g by using a digital balance, and height was measured to the nearest 0·5 cm by using a wall-mounted stadiometer. BMI was calculated as weight (kg)/height (m)^2^.

### Questionnaires

Questionnaires at baseline and at each consecutive week of the 6-week intervention period included the following: (1) the IBS Global Assessment of Improvement (IBS-GAI) score and (2) the IBS Symptom Severity Scale (IBS-SSS). The IBS-GAI asks participants: ‘Compared to the way you felt before you entered the study or the week before, have your IBS symptoms over the past 7 d been (1) ‘substantially worse’, (2) ‘moderately worse’, (3) ‘slightly worse’, (4) ‘no change’, (5) ‘slightly improved’, (6) ‘moderately improved’ or (7) ‘substantially improved’.’ The IBS-SSS contains five questions that measure, on a 100-point visual analogue scale, the severity of abdominal pain, the frequency of abdominal pain, the severity of abdominal distention, dissatisfaction with bowel habits and interference with the quality of life. All five components equally contribute to the score, yielding a theoretical range of 0–500, with a higher score indicating a worse condition. Previous studies have established that scores below 175 represent mild IBS symptoms, 175–300 represent moderate IBS symptoms and above 300 represent severe IBS symptoms^(^ ^9^ ^)^. The total severity score on the IBS-SSS was treated as the gold standard measure of IBS severity. In the study by Francis *et al.* ^(^ ^9^ ^)^, a decrease of 50 points correlated with improvement in clinical symptoms.

For the present study, we identified the subjects who responded to the intervention according to the main outcomes described above. As such, two different responder definitions were compared: (1) IBS-GAI, where a responder was defined as a patient whose symptoms were either ‘moderately improved’ or ‘substantially improved’ compared with the previous 6 weeks; (2) change in total IBS-SSS score from enrolment to 6 weeks of follow-up, where a respondent was classified as a patient whose overall symptom severity on the IBS-SSS changed ≥ 50 points.

### Characteristics of the wheat varieties

The mineral element content and secondary metabolite content were measured as described previously^(^ ^5^ ^)^.

### Blood measurements

Venous blood samples were taken from the subjects in the fasted state by the study physician and collected into evacuated plastic tubes (Vacutainer). Samples obtained by centrifuging at 3000 ***g*** for 15 min at 4°C were stored in aliquots at − 80°C until analysis. Lipid variables, blood glucose, serum electrolytes and liver enzymes were assessed by conventional methods. Pro- and anti-inflammatory cytokine levels were determined by using the Bio-Plex Cytokine Assay (Bio-Rad Laboratories, Inc.), according to the manufacturer's instructions.

### Statistical analysis

Statistical analysis was performed using the statistical package PASW 18.0 for Macintosh (SPSS, Inc.). All variables were checked for a normal distribution before data analysis. Data are expressed as arithmetic means and standard deviations for normally distributed variables and as medians and ranges for non-normally distributed data. The Mann–Whitney *U* test was used for testing the differences between the groups. A one-way ANOVA was used for testing the differences between the ancient and modern wheat. Non-normally distributed data were log-transformed, and further analysis was carried out with the transformed data. *Post hoc* Bonferroni correction was applied to account for multiple comparisons. The two interventions were analysed by taking into account both periods in the two groups of subjects at different stages. A general linear model for repeated measurements was used to compare the effect of the two different treatments. A model with adjustments for age and sex was used. Data for the general linear model are reported as geometric means with their standard errors. A *P* value < 0·05 was considered to indicate statistical significance.

## Results

### Characteristics of the wheat varieties

Flour and semolina were characterised for various mineral elements. The ancient semolina and flour varieties contained a significantly higher content of many minerals such as K, Fe, Mg, P, Se and Zn in comparison with the modern semolina and flour varieties (Table 1). Conversely, Ca content was significantly lower in the ancient semolina and flour varieties than in the modern counterparts. Total contents of polyphenols, carotenoids and flavonoids were also measured. Polyphenol and carotenoid contents were significantly higher in the ancient semolina and flour varieties than in the modern ones, while flavonoid content was significantly lower only in the modern flour variety.

**Table 1:** Composition of ancient and modern wheat (Mean values and standard deviations)

	Ancient wheat (semolina)	Modern wheat (semolina)		Ancient wheat (flour)	Modern wheat (flour)	
Variables	Mean	sd	Mean	sd	*P*	Mean	sd	Mean	sd	*P*
Ca (mg/kg)	216·1	28·8	356·7	15·1	0·03	206·9	4·1	339·4	20·5	0·01
Fe (mg/kg)	40·9	0·42	17·2	0·12	0·001	30·4	0·81	17·2	0·12	0·002
K (mg/kg)	3658·7	138·2	3237·5	52·4	0·05	3469·8	74·2	2153·4	80·6	0·003
Mg (mg/kg)	1373·9	43·9	806·5	32·4	0·005	1289·2	38·3	538·4	40·9	0·003
P (mg/kg)	3192·3	106·5	2494·5	16·9	0·01	3058·4	27·9	1708·1	15·9	< 0·0001
Se (mg/kg)	1·59	0·17	0·74	0·09	0·02	1·51	0·31	0·47	0·05	0·04
Zn (mg/kg)	28·3	1·26	20·4	0·63	0·02	25·8	0·36	17·3	0·72	0·04
Polyphenols (mg/g DM)	2·04	0·16	1·74	0·80	0·02	1·73	0·36	1·49	0·04	< 0·0001
Carotenoids (μg/g DM)	14·9	0·52	12·9	0·76	0·005	15·7	1·18	8·11	0·75	< 0·0001
Flavonoids (mg/g DM)	0·31	0·03	0·29	0·05	0·5	0·29	0·05	0·26	0·03	0·004

### Characteristics of the study population

A total of twenty subjects (seven males and thirteen females) with a median age of 35·5 (range 18–59) years were enrolled in the study. The average BMI was 22·3 (sd 4·1) kg/m^2^. Of these participants, three were current smokers and one was hypertensive under an optimal therapeutic control. Baseline questionnaires for evaluating the severity of symptoms related to IBS showed a moderate severity in all participants (total score for IBS-SSS 237 (sd 48·3), with a score ranging from 0 to 500). At the end of the intervention period, BMI did not change significantly with respect to baseline in both groups (data not reported).

### Changes in symptoms

After baseline evaluation, the study participants were randomised into two groups at the start of each respective dietary intervention period (either ancient or modern). No differences for demographic characteristics and severity of symptoms at baseline between the two groups of intervention were observed (data not reported).

**Fig. 1:**
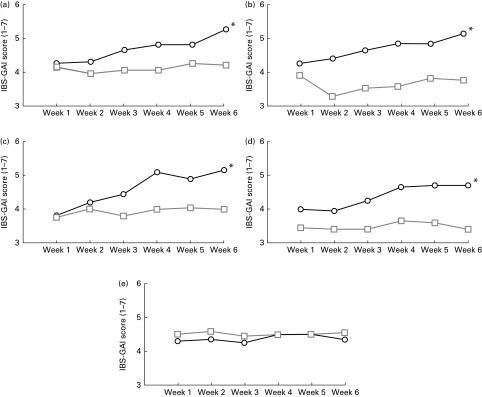
Changes in the irritable bowel syndrome-Global Assessment of Improvement (IBS-GAI) score (1 = worst and 7 = better) for (a) severity of abdominal pain, (b) severity of bloating, (c) satisfaction with stool consistency, (d) severity of tiredness and (e) severity of nausea in the ancient 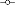 and modern 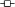 wheat-treated groups over a 6-week period. * Changes in the improvement of the different symptoms were significantly different from those observed in the modern wheat-treated group (*P*< 0·05).

To evaluate the changes in symptoms related to IBS over the entire study period, participants were asked to report the improvement and severity of each different symptom by using two different questionnaires (IBS-GAI and IBS-SSS). The IBS-GAI questionnaire showed that the ancient wheat products resulted in a significant trend of improvement for all the evaluated IBS symptoms except for nausea, using a general linear model adjusted for age and sex (Fig. 1). Moreover, the changes in the improvement for the different symptoms were also compared from baseline to the end of the intervention period, by showing a significant improvement in abdominal pain (score at baseline 4·25 (sd 1·07) *v*. score at week 6 5·25 (sd 1·62); *P*< 0·0001), bloating (score at baseline 4·30 (sd 1·30) *v*. score at week 6 5·20 (sd 1·58); *P*= 0·004), satisfaction of stool consistency (score at baseline 3·80 (sd 1·10) *v*. score at week 6 5·15 (sd 1·66); *P*< 0·0001), tiredness (score at baseline 4·0 (sd 0·56) *v*. score at week 6 4·70 (sd 1·22); *P*< 0·0001), but not nausea (score at baseline 4·30 (sd 0·87) *v*. score at week 6 4·35 (sd 1·21); *P*= 0·7) after consumption of the ancient wheat products. No significant difference was observed after consumption of the modern wheat products.

Subjects who responded to the intervention were defined as those who answered that, compared with 6 weeks ago, their symptoms were either ‘moderately improved’ or ‘substantially improved’. Of the total subjects, ten (50 %) were defined as responders for the symptom of abdominal pain after the ancient wheat intervention period, compared with only five (25 %) subjects after the control period. Similarly, ten (50 %) subjects reported to have improved abdominal bloating at the end of the ancient wheat intervention period, whereas only three (15 %) subjects reported a significant improvement after the intervention period with the modern wheat products, and twelve (60 %) subjects declared to have significantly improved satisfaction with bowel habits after the ancient wheat intervention period, compared with only four (20 %) subjects who reported an amelioration after the intervention period with the modern wheat products.

**Fig. 2:**
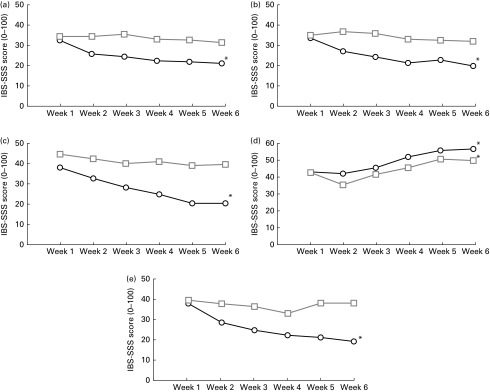
Changes in the irritable bowel syndrome-Symptom Severity Scale (IBS-SSS) for (a) abdominal pain, (b) frequency of abdominal pain, (c) bloating, (d) satisfaction with stool consistency and (e) interference with the quality of life in the ancient 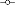 and modern 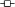 wheat-treated groups over a 6-week period. IBS-SSS score: (a, b, c and e) 0 = none and 100 = worst; (d) 0 = minimal and 100 = maximal. * Changes in the severity of the different symptoms were significantly different from those observed in the modern wheat-treated group (*P*< 0·05).

In addition, the severity of symptoms was also evaluated using a visual analogue scale (IBS-SSS). During the ancient wheat intervention period, participants experienced a significant decrease in the severity of all the symptoms evaluated (Fig. 2). The severity score of abdominal pain changed from 30·5 (sd 19·9) at baseline to 21·5 (sd 13·3) at week 6 (*P*= 0·001), the severity score of frequency of abdominal pain decreased from 35 (sd 27) at baseline to 20 (sd 11·2) at week 6 (*P*< 0·0001), the severity of bloating changed from 36·5 (sd 19·5) at baseline to 20·5 (sd 17) at week 6 (*P*< 0·0001), the satisfaction of stool consistency increased significantly from 44·5 (sd 17·9) at baseline to 58·5 (sd 18·9) at week 6 (*P*= 0·007) and the severity of interference with the quality of life reduced from 34 (sd 22·1) at baseline to 19·5 (sd 11·1) at week 6 (*P*< 0·0001).

By grouping subjects according to the total score on the IBS-SSS, it was evident that subjects who consumed the ancient wheat products reported an improved score at the end of the intervention period compared with those who consumed the control products (162 (sd 79·4) *v*. 230 (sd 101·7); *P*< 0·005). In particular, fourteen (70 %) subjects were defined as responders (i.e. improvement of 50 points of the total IBS-SSS score) after consumption of the ancient wheat products, compared with only five (25 %) patients after consumption of the modern wheat products.

### Modifications of biochemical and inflammatory parameters

Biochemical and inflammatory parameters were also evaluated at all the different study points. With regard to biochemical parameters, no significant difference was observed for all the investigated parameters after the intervention period with either the ancient or modern wheat products (Table 2). However, significant improvements in the circulating levels of pro-inflammatory cytokines were reported after the intervention period with only the ancient wheat products (Table 3). Decreases in the levels of the following cytokines were observed: IL-4 − 18·5 %; IL-6 − 36·2 %; IL-17 − 23·3 %; interferon-γ (IFN-γ) − 33·6 %; macrophage inflammatory protein-1β − 38·9 %; vascular endothelial growth factor − 23·7 %.

**Table 2:** Modifications of biochemical parameters* (Geometric mean values and interquartile ranges (IQR))

	Ancient wheat (pre)	Ancient wheat (post)		Modern wheat (pre)	Modern wheat (post)	
Variables	Mean	IQR	Mean	IQR	*P*	Mean	IQR	Mean	IQR	*P*
Blood glucose (mmol/l)	4·63	4·40–4·86	4·71	4·55–4·88	0·3	4·63	4·48–4·81	4·76	4·53–4·97	0·2
Total cholesterol (mmol/l)	4·77	4·37–5·17	4·69	4·39–4·98	0·3	4·73	4·41–4·97	4·84	4·49–5·20	0·1
LDL-cholesterol (mmol/l)	2·72	2·38–3·07	2·66	2·35–2·98	0·3	2·65	2·37–2·94	2·78	2·49–3·06	0·05
HDL-cholesterol (mg/l)	1·62	1·49–1·74	1·61	1·48–1·74	0·8	1·66	1·53–1·79	1·69	1·55–1·84	0·3
TAG (mmol/l)	0·91	0·73–1·09	0·93	0·78–1·08	0·7	0·96	0·78–1·14	1·05	0·76–1·22	0·5
Na (mEq/l)	140·5	139·9–141·1	140·7	140–141·3	0·7	140·8	140–141·6	140·6	139·6–141·5	0·6
K (mEq/l)	4·34	4·20–4·47	4·43	4·26–4·60	0·1	4·52	4·37–4·67	4·38	4·20–4·55	0·1
Mg (mg/l)	21·7	21·2–22·2	21·7	21·1–22·3	0·1	21·6	20·8–22·3	21·9	21·5–22·4	0·3

* General linear model adjusted for age and sex.

**Table 3:** Modifications of the inflammatory profile* (Geometric mean values and interquartile ranges (IQR))

	Ancient wheat (pre)	Ancient wheat (post)		Modern wheat (pre)	Modern wheat (post)	
Variables	Mean	IQR	Mean	IQR	*P*	Mean	IQR	Mean	IQR	*P*
IL-4 (pg/ml)	0·92	0·78–1·06	0·75	0·67–0·83	0·03	0·84	0·68–0·99	0·75	0·67–0·83	0·2
IL-6 (pg/ml)	3·56	2·27–4·86	2·27	1·46–3·08	0·02	3·49	1·61–5·38	3·06	1·93–4·20	0·5
IL-8 (pg/ml)	11·9	7·85–15·9	8·37	6·55–10·2	0·1	8·84	7·4–10·3	10·8	7·25–14·3	0·3
IL-10 (pg/ml)	1·30	0·15–2·5	1·46	0·92–1·99	0·9	1·52	0·90–1·74	1·44	0·29–2·38	0·6
IL-12 (pg/ml)	14·5	11·5–17·6	12·6	9·88–15·2	0·06	14·2	10·5–17·9	13·3	10–16·5	0·4
IL-17 (pg/ml)	8·68	6·31–11	6·66	4·56–8·76	0·03	8·27	6·01–10·5	7·16	4·78–9·54	0·4
IFN-γ (pg/ml)	26·5	17·5–35·6	17·6	14·4–20·8	0·03	24·6	15·9–33·2	20·2	14·7–25·6	0·2
IP-10 (pg/ml)	503·8	401·7–606·1	630·8	413·6–847·9	0·3	480·4	372·5–588·3	507·7	370·5–644·9	0·7
MCP-1 (pg/ml)	38·6	28·1–49·1	23·6	18·9–28·3	0·009	39·4	25·3–53·4	25·4	16·7–37·1	0·05
MIP-1β (pg/ml)	98·8	81·9–115·6	98·1	75·9–120·3	0·9	96·5	79–113·9	105·3	75·4–135·2	0·4
TNF-α (pg/ml)	2·56	1·18–4·41	2·42	1·02–4·01	0·7	2·34	1·22–3·45	2·47	1·39–3·50	0·3
VEGF (pg/ml)	69·7	47·3–92·2	53·2	35·2–71·2	0·02	65·9	37·9–94·1	64·2	44·8–83·6	0·1

IFN-γ, interferon-γ; IP-10, interferon-γ-induced protein-10; MCP-1, monocyte chemotactic protein-1; MIP-1β, macrophage inflammatory protein-1β; VEGF, vascular endothelial growth factor.

* General linear model adjusted for age and sex.

## Discussion

The aim of the present study was to assess the effect of a replacement diet with *T. turgidum* subsp. *turanicum* (ancient) wheat products on both IBS symptom improvement/severity and inflammatory/biochemical parameters in participants classified as having moderate IBS. After consumption of ancient wheat products, patients experienced a significant global improvement in the extent and severity of symptoms related to IBS such as bloating, abdominal distention, abdominal pain and frequency, tiredness and satisfaction of stool consistency, with a consequent improvement in the quality of life. Conversely, no significant improvement was noted during the modern wheat intervention period. To the best of our knowledge, the present study is the first one to show that a replacement diet with an ‘ancient wheat product’ is able to bring about symptom relief in such a relevant syndrome. Satisfactory relief is considered useful in IBS trials^(^ ^10^ ^)^, as improvements that result in improvements to the overall quality of life are ultimately the most important benefits in treating such type of gastrointestinal disorders^(^ ^11^ ^)^.

Over recent years, studies have suggested that IBS symptoms in one-quarter of all patients might be influenced by diet^(^ ^3^ ^)^. Actually, the role of diet in the management of this relevant syndrome is of increasing interest in clinical practice. Some patients with IBS respond well to a gluten-free diet; thus, in the evaluation of exclusion diets, wheat has been found to play a relevant role, and the first recommended dietary change for IBS patients is the elimination of wheat products from the diet, mainly due to the possible ‘toxic’ effect of gluten^(^ ^3^ ^,^ ^12^ ^)^.

As a result, this has contributed to an increased number of non-coeliac subjects who consume gluten-free products without any precise medical advice or indication. In line with the increasing interest in wheat products as possible mediating factors for IBS, we have recently carried out some studies on ancient grain varieties, showing that these varieties contain different nutritional characteristics with respect to modern varieties^(^ ^4^ ^,^ ^5^ ^)^. Among them, *T. turgidum* subsp. *turanicum* has attracted increasing interest for the general population and especially for patients suffering from IBS. Although such ancient wheat does contain gluten, the healthier nutritional profile of this ancient grain variety, in comparison with that of the modern variety, resulted in improvements in mineral, antioxidant and anti-inflammatory properties, which were similarly projected to result in beneficial effects for patients with IBS^(^ ^5^ ^)^. In our previous study, the total soluble dietary fibre component did not vary between the ancient wheat and the modern wheat^(^ ^5^ ^)^. Individual constituents of the soluble fibres that potentially alleviate IBS symptoms, such as fructo-oligosaccharides (oligo-fructans) which have been reported to reduce abdominal pain, constipation and diarrhoea, have yet to be examined in the ancient wheat^(^ ^3^ ^)^. In this preliminary double-blinded dietary intervention study, we were able to demonstrate that a 6-week period of a replacement diet with products made from ancient wheat is useful in improving symptoms in patients with IBS.

Along with improvements in the frequency and severity of symptoms related to IBS, a significant decrease in the circulating levels of pro-inflammatory cytokines (IL-6, IL-17, IFN-γ, monocyte chemotactic protein-1 and vascular endothelial growth factor), following a replacement diet with ancient wheat products, was observed, thereby verifying our working premise, formulated after noting significant improvements in the inflammatory profile of a healthy population^(^ ^5^ ^)^. This is first study that provided evidence for the potential role of a ‘staple food’ towards improving circulating levels of cytokines in parallel with improvements in IBS symptoms.

The positive and significant improvements in IBS symptoms associated with changes in the profile of pro-inflammatory cytokines lend support to IBS as being a low-grade inflammation condition^(^ ^6^ ^)^. In particular, the significant decrease in IL-6 levels and improvements in IBS symptoms corroborate previous findings pointing to IL-6 as the most potentially interesting cytokine biomarker of IBS^(^ ^6^ ^,^ ^13^ ^)^. Studies demonstrating increased baseline IL-6 levels in IBS patients *v*. controls^(^ ^7^ ^,^ ^8^ ^,^ ^14^ ^,^ ^15^ ^)^ as well as the cholinergic-mediated (brain–gut axis neurotransmitter) increased expression of IL-6 associated with increased abdominal pain/discomfort and bloating have provided evidence for pro-inflammatory IL-6 responses in IBS patients^(^ ^8^ ^)^. Most recently, a crosstalk between IL-6 and the stress peptide, corticotrophin-releasing factor, has offered more insight into the mechanisms underlying IBS symptoms during periods of stress^(^ ^13^ ^)^, which is experienced by almost two-thirds of all patients suffering from IBS^(^ ^16^ ^)^. Elevated levels of IL-6 and TNF-α have been shown to be associated with both increased tiredness and gut inflammation in both animal models and human subjects^(^ ^17^ ^)^. Interestingly, participants in the present study reported an improvement not only in the degree of abdominal pain and bloating but also in the level of tiredness after consumption of the ancient wheat products.

Apart from IL-6, potentially the most intriguing cytokine, the levels/responses of other cytokines were shown to be varied across different studies^(^ ^7^ ^)^. Overall, the most relevant findings cited in some of the studies have reported increased levels of IL-8 and TNF-α, associated with IBS patients (and not with controls), as well as significantly decreased levels of the anti-inflammatory cytokine IL-10^(^ ^7^ ^,^ ^18^ ^,^ ^19^ ^)^. No significant effect on the levels of IL-8 and TNF-α was reported in the present study, although a trend in reduction levels of such cytokines was evident. Given that IL-10 is reputed to be lower in patients suffering from IBS, it was interesting to note that the baseline level of the anti-inflammatory cytokine IL-10 was five to seven times lower in IBS patients of the present study, compared with that in control (non-IBS) subjects in a previous study of our group^(^ ^5^ ^)^.

A significant decrease in the levels of IFN-γ, monocyte chemotactic protein-1, IL-17 and vascular endothelial growth factor was also observed in the present study only after ingestion of the ancient wheat products. Increased IFN-γ levels might reduce serotonin levels with subsequent dysregulation of gastrointestinal motility and secretion in IBS patients^(^ ^15^ ^)^, hence it is of relevance that an improvement in IBS symptoms was associated with a decrease in the levels of IFN-γ. Increased monocyte chemotactic protein-1 levels have been reported as a characteristic in patients with predominantly diarrhoea-type IBS^(^ ^20^ ^)^. Interestingly, in the present study, a significant reduction in the levels of IL-17, IFN-γ and vascular endothelial growth factor after consumption of ancient wheat products was observed in patients with moderate IBS, although such cytokines have been reported to be mostly associated with more severe inflammatory bowel diseases^(^ ^7^ ^,^ ^21^ ^)^.

While the present study demonstrates an improvement in the inflammatory profile, the specific role of cytokines in IBS is not fully understood, and, to date, there are no unifying trends in cytokine profile changes among the various studies conducted^(^ ^7^ ^)^. Potential reasons include the small sample sizes in the reported studies where the entire spectrum of cytokines was often not measured^(^ ^7^ ^)^. Diet provides a variety of nutrients as well as an array of functional constituents (flavonoids and soluble fibres) that modulate inflammatory processes. No single functional constituent, responsible for improvements in the inflammatory profile, has yet been identified in ancient wheat products. Probably, the beneficial efforts are attributable to synergistic effects within the spectrum of various functional constituents present.

No effects on biochemical parameters were observed in the present study after a replacement diet with ancient wheat products. In a study by Halpert *et al.* ^(^ ^22^ ^)^ aimed at studying patients' perceptions about IBS, it was observed that more than 60 % of patients were careful about diet and keenly interested in understanding what foods to eat. Generally, patients suffering from IBS are ‘more’ diet conscious, and a lack of change in biochemical parameters (and taking into consideration the BMI) may reflect the interest in diet shown by the present participants^(^ ^3^ ^)^.

Although the present results are promising, the number of participants (twenty in total) represents the major limitation of the present study. Further and larger studies need to be conducted before drawing any firm conclusion on the effects of such foods in IBS. Before initiating the trial, all subjects were instructed by physicians and by an expert dietitian to maintain their usual lifestyle habits. Although it is never possible to implement such a check, this was not viewed as a constraint of the present study as all participants ingested both ancient and modern wheat products, and were acutely conscious of reporting their symptoms on a weekly basis. Moreover, their respective BMI did not change over the course of the study. The female:male ratio in the studied patients is also representative of the greater female prevalence with this disorder^(^ ^3^ ^,^ ^16^ ^)^.

In conclusion, the present study is the first one showing an association between decreased circulating levels of pro-inflammatory cytokines and improvements in important IBS symptoms such as reduced abdominal pain, bloating, tiredness and interference with the quality of life. Moreover, the present study shows that a replacement diet with a wheat grain product having functional properties is potentially effective in improving IBS symptoms. Further and larger studies are needed to confirm this dietary approach that could be of clinical interest for the large population of patients suffering from this syndrome.
